# Air processed Cs_2_AgBiBr_6_ lead-free double perovskite high-mobility thin-film field-effect transistors

**DOI:** 10.1038/s41598-022-06319-z

**Published:** 2022-02-14

**Authors:** Gnanasampanthan Abiram, Fatemeh Heidari Gourji, Selvakumar Pitchaiya, Punniamoorthy Ravirajan, Thanihaichelvan Murugathas, Dhayalan Velauthapillai

**Affiliations:** 1 Department of Physics, Faculty of Science, University of Jaffna, Jaffna, 40000 Sri Lanka; 2 Department of Computer Science, Electrical Engineering and Mathematical Sciences, Western Norway University of Applied Sciences, Inndalsveien 28, 5063 Bergen, Norway

**Keywords:** Chemistry, Energy science and technology, Materials science, Nanoscience and technology

## Abstract

This study focuses on the fabrication and characterization of Cs_2_AgBiBr_6_ double perovskite thin film for field-effect transistor (FET) applications. The Cs_2_AgBiBr_6_ thin films were fabricated using a solution process technique and the observed XRD patterns demonstrate no diffraction peaks of secondary phases, which confirm the phase-pure crystalline nature. The average grain sizes of the spin-deposited film were also calculated by analysing the statistics of grain size in the SEM image and was found to be around 412 (± 44) nm, and larger grain size was also confirmed by the XRD measurements. FETs with different channel lengths of Cs_2_AgBiBr_6_ thin films were fabricated, under ambient conditions, on heavily doped p-type Si substrate with a 300 nm thermally grown SiO_2_ dielectric. The fabricated Cs_2_AgBiBr_6_ FETs showed a p-type nature with a positive threshold voltage. The on-current, threshold voltage and hole-mobility of the FETs decreased with increasing channel length. A high average hole mobility of 0.29 cm^2^ s^−1^ V^−1^ was obtained for the FETs with a channel length of 30 µm, and the hole-mobility was reduced by an order of magnitude (0.012 cm^2^ s^−1^ V^−1^) when the channel length was doubled. The on-current and hole-mobility of Cs_2_AgBiBr_6_ FETs followed a power fit, which confirmed the dominance of channel length in electrostatic gating in Cs_2_AgBiBr_6_ FETs. A very high-hole mobility observed in FET could be attributed to the much larger grain size of the Cs_2_AgBiBr_6_ film made in this work.

## Introduction

In recent years, Perovskite thin-film field-effect transistors (FETs) have been extensively studied for a variety of applications such as photo FET^1^, light-emitting FET^2^, photo detectors^3^ and ferroelectric RAM^4^. Even though a huge number of Perovskite materials were studied for solar cell applications, only quite a few Perovskite materials have been studied for FET applications. Hybrid organic–inorganic halide Perovskites like methylammonium lead iodide (CH_3_NH_3_PbI_3_) have been the most dominant semiconducting channel materials used in the studies of Perovskite thin-film FETs^5^. All inorganic double perovskite material, Cs_2_AgBiBr_6_ is considered to be one of the promising alternative non-toxic and highly stable materials in the family of perovskites exhibiting a long charge carrier lifetime, high charge carrier mobility in single crystals, and effective charge carrier masses comparable to those of lead-based organic–inorganic perovskite semiconductors^6–9^. Recent theoretical investigations suggest that the Cs_2_AgBiBr_6_ material is a potential candidate for PV, photocatalytic and x-ray detector applications^10–12^. However, experimental limitations in fabricating large-grained and highly phase pure Cs_2_AgBiBr_6_ are the main bottlenecks^13–15^. Cs_2_AgBiBr_6_ displays superior stability towards ambient environments and has an indirect bandgap between 1.9 and 2.3 eV. Despite the eco-friendliness and long-term stability Cs_2_AgBiBr_6_ possess, the large indirect bandgap and poor light absorption ability of the material have restricted their applicability in solar cell technologies. The highest reported efficiency of Cs_2_AgBiBr_6_ based solar cells to date is 3.11%, whereas the highest certified efficiency reported for hybrid organic–inorganic lead-halide perovskite-based solar cells is around 25.5%^13,16^. Nevertheless, despite the high trap densities in Cs_2_AgBiBr_6_, due to their long charge-carrier lifetimes (larger than a microsecond) owing to the shallow nature of the majority of traps, diffusion lengths exceeding 1 µm and long-term environmental stability than organic–inorganic lead-halide perovskites, make it a promising material for a wide range of applications such as light-emitting diodes (LEDs), radiation detectors, photodetectors, photocatalysts, sensors and in neuromorphic computing^17–20^.

This study focuses on investigating the structural phase purity and morphological formation of solution-processed Cs_2_AgBiBr_6_ double perovskite thin films. Cs_2_AgBiBr_6_ double perovskite has primarily been studied for solar cells applications^21^ until recent study on its application for FET by Li et al.^22^ Cs_2_AgBiBr_6_ double Perovskite thin-film has been served as channel which is studied based on the existence of grains and grain boundaries of interlayers^17,22^. Li et al*.*, studied the role of grain boundaries on charge carrier and ion transport in Cs_2_AgBiBr_6_ Perovskite thin films through FET^22^. By tailoring the grain size within the range of 70 nm to 110 nm, they studied the role of the grain boundaries in ion transportation with the support of cathodoluminescence imaging. In this work, we employ Cs_2_AgBiBr_6_ material with larger grain size as the semiconducting channel in a FET. We report the very first Cs_2_AgBiBr_6_ FET ever fabricated in air processing under ambient conditions. The gating mechanism of the fabricated Cs_2_AgBiBr_6_ FETs is discussed by analysing the electronic properties of FETs by varying channel lengths. The Cs_2_AgBiBr_6_ film was fabricated with a grain size of 412 (± 44) nm and its electronic properties were evaluated. The field hole mobility of the fabricated Cs_2_AgBiBr_6_ was also calculated in this work. The average hole mobility of the Cs_2_AgBiBr_6_ channel is found to have a remarkably high value of 0.29 cm^2^ s^−1^ V^−1^ whereas the only previously reported values for all inorganic Cs_2_AgBiBr_6_ were in the order of 10^–3^ cm^2^ s^−1^ V^−1^. We conclude that the enhanced mobility could be attributed to the larger grain size and high-quality thin film produced by recrystallization of Cs_2_AgBiBr_6_ crystals during the thin film deposition.

## Results and discussions

**Figure 1:**
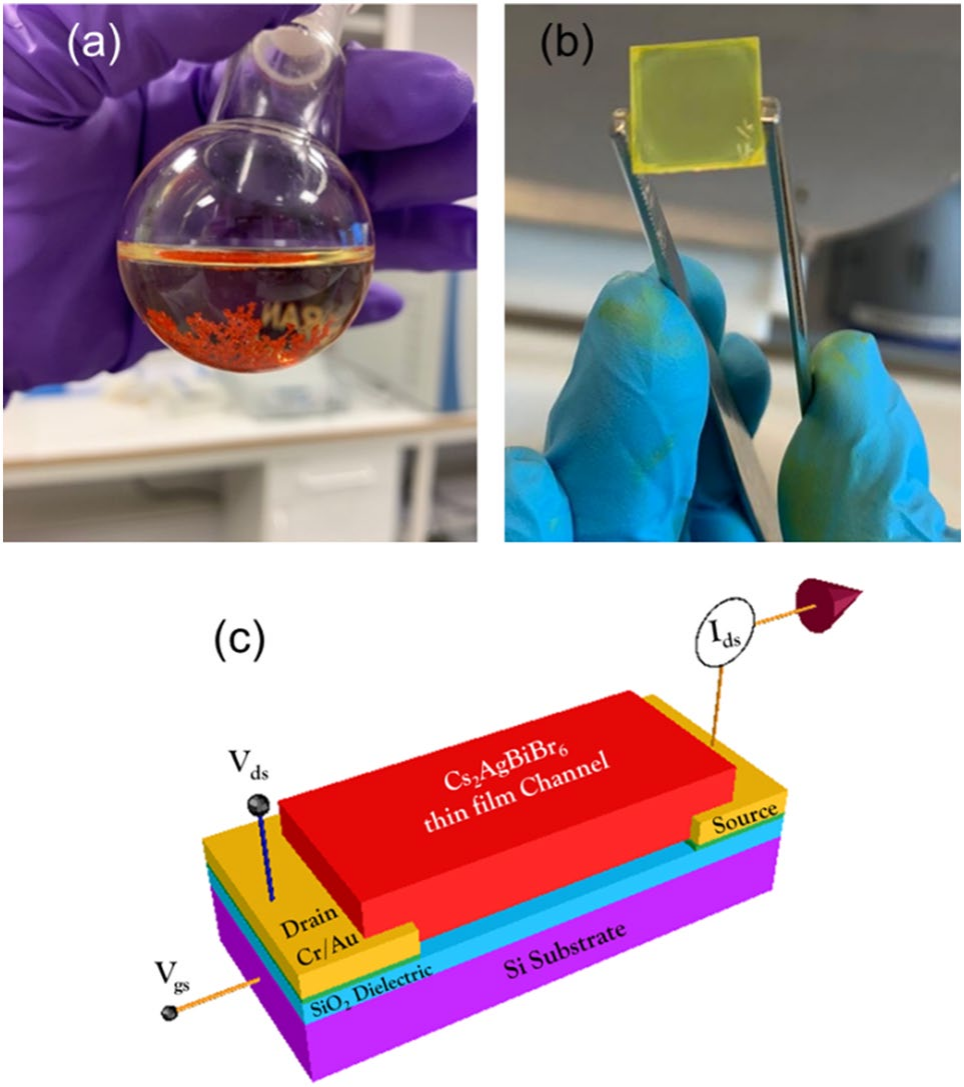
Photograph of (**a**) grown Cs_2_AgBiBr_6_ crystal in a conical flask and (**b**) Cs_2_AgBiBr_6_ thin film deposited glass surface. (**c**) Schematic of the Cs_2_AgBiBr_6_ FET fabricated in our work and circuit connections made for electrical characterizations.

The Cs_2_AgBiBr_6_ thin film was fabricated via a two-step route. Firstly, the Cs_2_AgBiBr_6_ crystals were grown as described in the Methods section (the grown crystals are illustrated in Fig. 1a) and then the precursor for spin coating was prepared by dissolving the crystals in dimethyl sulfoxide (DMSO). Thin films were fabricated by spin coating and subsequently calcined at 280 °C for 10 min (Fig. 1b). The Cs_2_AgBiBr_6_ FET devices with bottom gate bottom contact (BGBC) device structure were fabricated as shown in Fig. 1c. The BGBC structure was used in our study as it has shown better device performance and is one of the most studied device structures^17,22,23^.

**Figure 2:**
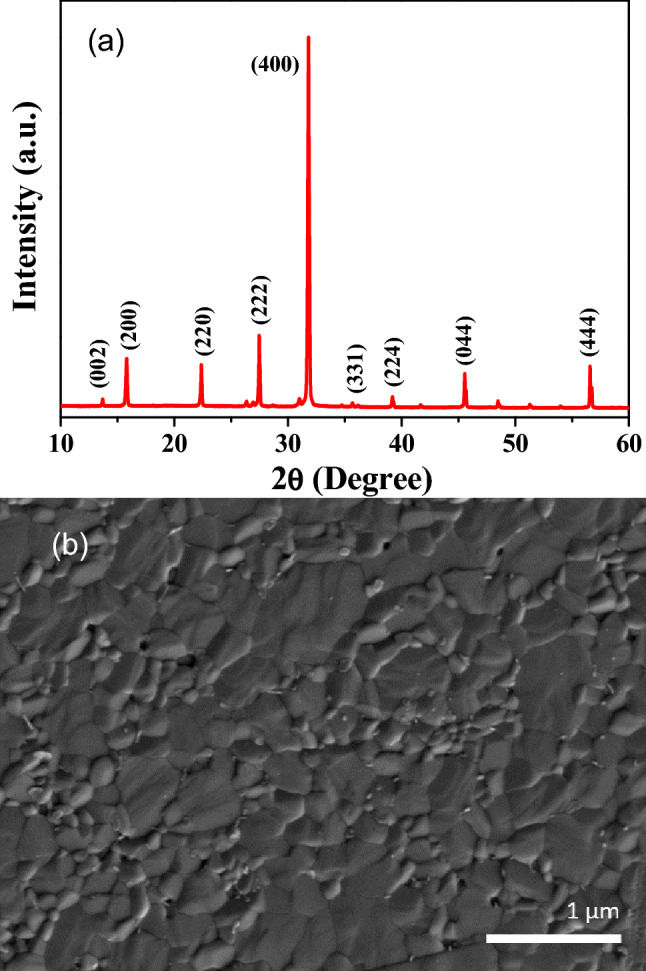
(**a**) XRD pattern and (**b**) SEM image of the prepared Cs_2_AgBiBr_6_ double perovskite thin films.

Prior to device fabrication, XRD measurements were carried out to confirm the structural and phase purity of the spin-coat deposited Cs_2_AgBiBr_6_ double perovskite thin film and the obtained result is depicted in Fig. 2a. As to avoid two major unwanted intermediate phases of Cs_3_Bi_2_Br_9_ (reflection at 12.8° and 30.9°) and AgBr compounds (reflection at 44.2°), the film was annealed at 280 °C for 5 min which was found to be the best condition (285 °C) for the formation of phase-pure double perovskite structure during the film formation^24^. From the observed XRD results, all the obtained major peaks located at 13.63°, 15.73°, 22.34°, 27.41°, 31.77°, 35.56°, 39.23°, 45.56°, and 56.53° attribute to the reflections of Cs_2_AgBiBr_6_ having the plane values of (002), (200), (220), (222), (400), (331), (224), (044), and (444), all the peaks correlate with the standard JCPDS (File number: 01-084-8699) data and are also in good agreement with the previous reports on double perovskite Cs_2_AgBiBr_6_ materials^25–27^. There were no specific secondary residual reflections of Cs_3_Bi_2_Br_9_ or AgBr, that confirm the phase-pure crystalline nature of the prepared Cs_2_AgBiBr_6_ thin film as depicted in the inset of Fig. 2a. In addition, the average crystallite size for the prepared Cs_2_AgBiBr_6_ sample was calculated using the Debye–Scherrer formula from the predominating peak signal found at 2θ = 31.77° related to the (400) plane. The observed full width at half maximum (FWHM) of the spin-coated Cs_2_AgBiBr_6_ film was found to be very low, and the grain size was measured as 485 nm. We also calculated the grain size using SEM image. Figure 2b depicts the polygonal grain structured surface morphological appearance for the spin-deposited Cs_2_AgBiBr_6_ double perovskite thin-film observed from the SEM analysis. The average grain size of the film was also calculated by analysing the statistics of grain size in the SEM images using ImageJ software. Accordingly, the calculated result benchmarks the average grain size from the surface morphology was found to be 412 (± 44) nm. The grain size is much less than the shortest channel length (30 µm) of the FET we tested. The significantly larger grain size achieved up to micro-size (average ~ 412 nm) could be mainly due to recrystallization of phase pure Cs_2_AgBiBr_6_ and the structural reconstruction of Cs_2_AgBiBr_6_ grains by the higher annealing temperature (~ 285 °C) process^14^. Remarkably, the achieved uniform and larger grain size all over the surface of the thin films with reduced grain boundaries were found to be the key factor in determining efficient charge-carrier and ion transport as reported in various semiconducting device applications^14,22,28^.

**Figure 3:**
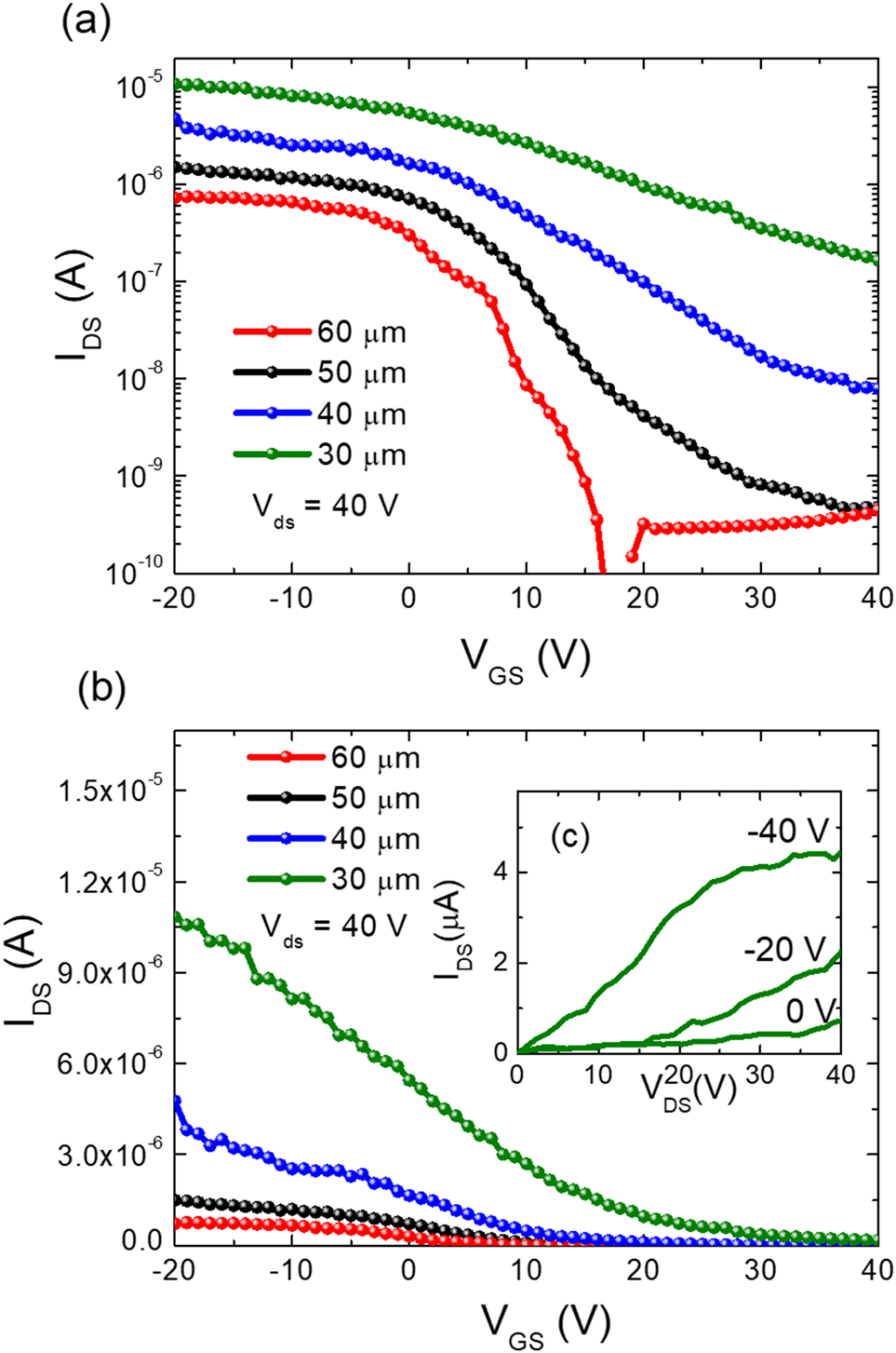
Transfer characteristic curves of Cs_2_AgBiBr_6_ FET with channel lengths of 30 µm, 40 µm, 50 µm and 60 µm (**a**) semi-log scale and (**b**) linear scale, and (**c**) the group of output characteristic curves of Cs_2_AgBiBr_6_ FET with channel length of 30 µm.

The transfer characteristics of the fabricated Cs_2_AgBiBr_6_ thin film FETs with channel lengths of 30, 40, 50 and 60 µm were studied within the gate voltage (V_gs_) range of − 20 V to 40 V under the applied constant source-drain (V_ds_) voltage of -40 V. The transfer curves with the logarithmic and linear current axis are shown in Fig. 3a and b respectively. From the transfer curves, we analysed the electronic properties of the Cs_2_AgBiBr_6_ material and the role of channel length in gating. The maximum observed current from the transfer characteristics curve in Fig. 3a is identified as the on-current of the Cs_2_AgBiBr_6_ FET devices. The minimum necessary gate voltage to turn on the Cs_2_AgBiBr_6_ FET device is the threshold voltage V_th_. The intercept of the extension line drawn in the linear region of I_ds_ vs V_gs_ curve and V_gs_ axis in the transfer characteristics curve in Fig. 3b gives the threshold voltage of Cs_2_AgBiBr_6_ FET. The output characteristic curves of the FET with 30 µm channel was shown in Fig. 3c as an inset of Fig. 3b. The output current I_DS_ against the voltage V_DS_ at a gate voltage of − 40 V saturates and shows an ideal output curve. Therefore, we used the saturation mobility equation for extracting the field effect hole-mobility. The field effect hole-mobility $\left({\upmu }_{\mathrm{h}}\right)$ values were calculated using the following equation^22,29^,

$${\upmu }_{\mathrm{h}}=\frac{2L}{\mathrm{wC}}{\left(\frac{\mathrm{d}(\sqrt{{\mathrm{I}}_{\mathrm{ds}}})}{{\mathrm{dV}}_{\mathrm{gs}}}\right)}^{2}$$

where $\mathrm{L}$ is the channel length (30 to 60 µm), $\mathrm{w}$ is channel width (2 mm), $\mathrm{C}$ is the effective capacitance of the dielectric material which is 300 nm thickness of SiO_2_ (7.23 × 10^–9^ Fcm^−2^) and $\frac{\mathrm{d}(\sqrt{{\mathrm{I}}_{\mathrm{ds}}})}{{\mathrm{dV}}_{\mathrm{gs}}}$ is the slope of the $\sqrt{{\mathrm{I}}_{\mathrm{ds}}}$ vs ${\mathrm{V}}_{\mathrm{gs}}$ graph in the linear region.

**Figure 4:**
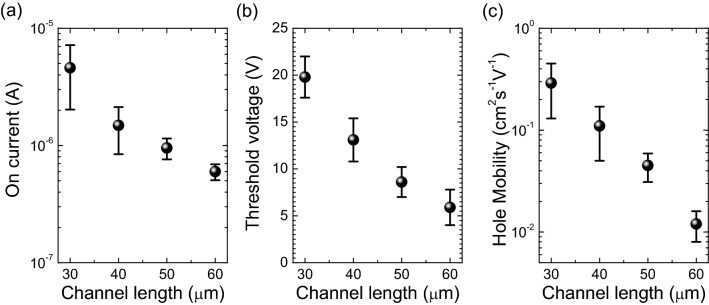
(**a**) On current, (**b**) threshold voltage and (**c**) hole mobility of the Cs_2_AgBiBr_6_ Perovskite FETs with different channel lengths of 30 µm, 40 µm, 50 µm and 60 µm.

The variations of on-current, threshold voltage and hole-mobility of Cs_2_AgBiBr_6_ FET along with the channel lengths of 30 µm, 40 µm, 50 µm and 60 µm are shown in Fig. 4. The variation of on-current values for the Cs_2_AgBiBr_6_ FETs with varying channel lengths are shown in Fig. 4a. For 30 µm long Cs_2_AgBiBr_6_ channel in FET, the average on-current is about 5 µA and it logarithmically decreases with the increasing channel length. For 60 µm long Cs_2_AgBiBr_6_ channel FET, the average on current reaches the value of 0.5 µA, which is an order of magnitude lower than the on current through the 30 µm channel. The variation of the threshold voltages of the Cs_2_AgBiBr_6_ FETs with different channel lengths are shown in Fig. 4b. There is a clear indication that the threshold voltages decrease as the channel lengths increase from 30 µm to 60 µm. Likewise, the hole-mobility of the Cs_2_AgBiBr_6_ channel also reduces with the increment of the Cs_2_AgBiBr_6_ channel lengths (Fig. 4c). High average hole mobility of 0.29 cm^2^ s^−1^ V^−1^ was obtained for the FETs with a channel length of 30 µm, and it got reduced by an order of magnitude (0.012 cm^2^ s^−1^ V^−1^) when the channel length increased to 60 µm. High on-current in the shortest channel length FET can be explained by the shortest resistive path that the change carriers had to travel through. Decreasing on-current with increasing channel length is an indication of increasing channel resistance with channel length. When the channel length is doubled (30 to 60 µm), the on-current decreases by an order of magnitude.

## Discussion

To study the role of channel length in electrical conduction and gating, we fitted the on current and mobility vs channel length curves with Origin 8.5 software. The on current (I_on_) vs channel length (L) curve was found to follow a power relationship of I_on_ = aL^b^ with an R^2^ value greater than 0.97. The fitting equation results in a ‘b’ value of − 2.975 (± 0.27), which indicates that the on current is proportional to the third power of the channel length L. Meanwhile, the hole mobility of the FETs also showed a power fit (with an R^2^ value of greater than 0.94) with a ‘b’ value of − 4.59 (± 0.42) indicating that the mobility is nearly proportional to the fifth power of the channel length (Fig. 4c). The power fit of both on current and mobility confirms the strong dependence of electrical conductance and hole mobility in the channel length. Changing the channel length to study the charge transport of (C_6_H_5_C_2_H_4_NH_3_)_2_SnI_4_ perovskite channel was previously done by Matsushima et al.^30^ They used a sequence of channel lengths varying from 45 to 750 mm to study the intrinsic charge transport properties of (C_6_H_5_C_2_H_4_NH_3_)_2_SnI_4_ perovskites. They observed hole and electron mobilities to increase with increasing channel length and to saturate after a particular channel length is reached. The increased mobility with increasing channel length is an indication of scatter free conduction and gating in the material^30^. However, as per our observation, in the Cs_2_AgBiBr_6_ perovskite FETs, the mobility decreased with increasing channel length. Hence, it is confirmed that the gating of these Cs_2_AgBiBr_6_ FETs is not dominated by the field modulated charge carriers in the channel. The strong dependence of on current in the channel length also confirms that the gating or conduction does not depend on the Schottky junctions formed at the electrode-channel junction.

It should be noted that the electrical conductance in the Cs_2_AgBiBr_6_ thin film is dominated by the grain boundaries^22^. From the power fits of on current and hole mobility, and high gating in the longest channel FETs, one can confirm the role of grain boundaries in the charge transfer and gating in Cs_2_AgBiBr_6_ FETs. It is noted that the threshold voltage of the Cs_2_AgBiBr_6_ FETs linearly decreased with increasing channel length and always was positive within the channel length range of 30 to 60 µm. Positive threshold voltage also confirms that the majority change carriers of the Cs_2_AgBiBr_6_ FET are holes when the gate bias is not applied^31–34^. The negative shift in threshold voltage with increasing channel length is a significance of reduced effective hole concentration with increasing channel length which is opposite to the observations made by Matsushima et al. on (C_6_H_5_C_2_H_4_NH_3_)_2_SnI_4_ perovskite FETs^30^. This also shows that the electron and hole transport can be hindered by the grain boundaries during the transport. It is also noted that the grain boundary is an important contributor to the recombination losses^20^. Particularly, the lowering on-currents and mobilities with increasing channel length is a strong indication of the role of charge scattering at the grain boundary regions. This observation is in accordance with the observations reported by Li et al. Hence, it can be concluded that the grain boundaries are creating blockades during the charge transfer through the channel. The longer the channel, the more grain boundaries have to be tunnelled and hence the conductivity gets heavily decreased. Moreover, the applied gate voltage hugely influences the barrier height at the grain boundaries and hence the charge career tunnelling across the grain boundaries. Thus, we can conclude that the grain boundaries are the conduction and gating hotspots in the Cs_2_AgBiBr_6_ FETs and the main reason for the lower on-current and mobilities in the 60 µm channel FETs.

Cs_2_AgBiBr_6_ FET studies are in the initial stage and it is important to have a collation with the only published report on Cs_2_AgBiBr_6_ FET. The characteristics of the device made in this study are thus compared with the previous work on Cs_2_AgBiBr_6_ FET in Table 1 ^22^. The hole mobility of the Cs_2_AgBiBr_6_ channel is low as 1.5 × 10^–3^ cm^2^ V^−1^ s^−1^ in the previously reported study. In our fabricated Cs_2_AgBiBr_6_ FETs, we found a very high average hole mobility of 0.29 cm^2^ V^−1^ s^−1^ which is two orders of magnitude higher than the previously reported work. This could be attributed to the much larger grain size of our Cs_2_AgBiBr_6_ film, which is about four times bigger than the grain size reported earlier. Though we used the air processing method to fabricate the device, the basic properties like majority carrier and ground state conduction characteristics of our device do not differ notably from the mentioned previous study, where N_2_ environment was used in fabrication with a channel length of 20 µm and width of 1 mm.

**Table 1:** Comparison of figure of merits of our Cs_2_AgBiBr_6_ FET with the existing literature.

	In the literature^22^	In our work
Device structure	BGBC	BGBC
Fabrication environment	N_2_	Air
Channel dimension	20 µm × 1 mm	30 µm × 2 mm
Average grain size	~ 110 nm	~ 412 nm
Hole mobility (at 300 K)	15 × 10^–4^ cm^2^ V^−1^ s^−1^	0.29 cm^2^ V^−1^ s^−1^
V_ds_	− 60 V	− 40 V
ON current	5.5 × 10^–7^ A	4.12 × 10^–6^ A

## Conclusions

A FET with a lead-free, all-inorganic Cs_2_AgBiBr_6_ double perovskite thin film channel was successfully demonstrated. The Cs_2_AgBiBr_6_ thin film was fabricated by recrystallizing the pre-prepared Cs_2_AgBiBr_6_ solution followed by annealing at 285 °C, that enhanced the grain growth and resulted with a maximum grain size of 412 (± 44) nm. The channel length of the FET strongly influenced the on current, threshold voltage and hole mobility of the FETs. All three parameters were reduced with increasing channel length. The strong dependency of on current and mobility on the channel length confirmed the dominance of grain boundaries in the electrostatic gating in Cs_2_AgBiBr_6_ thin film FETs. The lower mobility and on-current in the longer channel FET can be attributed to the increased charge scattering at the effectively larger number of grain boundaries of the longest Cs_2_AgBiBr_6_ thin film. The maximum mobility of 0.29 (± 00.07) cm^2^ V^−1^ s^−1^ observed in the Cs_2_AgBiBr_6_ FETs with 30 µm channel was two orders of magnitude higher than the previously reported values for hole mobility of the same material due to the much larger grain size obtained for Cs_2_AgBiBr_6_ thin film in our study.

## Experimental methods

### Synthesis of Cs_2_AgBiBr_6_

The Cs_2_AgBiBr_6_ double perovskite crystals were synthesized through the solution-based process as reported elsewhere^35,36^. The detailed process are as follows: 2 mmol of CsBr (426 mg, 99.5%, Alfa Aesar), 1 mmol of AgBr (186 mg, 99.9%, Alfa Aesar) and 1 mmol of BiBr_3_ (446 mg, 99.99%, Alfa Aesar) metal salts were sequentially added and dissolved in 9 ml of hydrobromic acid (HBr, 48%, Merck) under magnetic stirring. Then, the as-prepared solution, moved to a round bottom flask and placed in a silicon oil bath under reflux reaction. The silicon oil gradually heated up to 120° and held for 2 h with gentle stirring of the solution. Thereafter, the solution smoothly was cooled down to room temperature with the rate of 5°/h. Finally, the well-grown (as shown in Fig. 1a) Cs_2_AgBiBr_6_ double perovskite crystals were collected by filtering and washed three times with absolute ethanol and dried overnight in vacuum oven, and the crystals could be grounded to obtain in powder form using pestle and mortar.

### Fabrication of Cs_2_AgBiBr_6_ FET

The Cs_2_AgBiBr_6_ FETs were fabricated on a 300 nm SiO_2_ grown heavily doped Si (SiO_2_/Si) substrate (Ossila Ltd, United Kingdom). The source and drain electrodes were deposited on top of the SiO_2_ dielectric layer by using successive thermal evaporation of 5 nm Chrome and 50 nm gold, with the channel lengths of 30 µm, 40 µm, 50 µm and 60 µm using Edwards E306 thermal evaporator under high vacuum of 10^–5^ mTorr^31,33^. The 5 nm Chrome was deposited as a sacrificial layer to assist the adhesiveness of gold on the SiO_2_ surface^31,33^. The pre synthesized Cs_2_AgBiBr_6_ crystals were dissolved in 250 µl of DMSO (100 ml, anhydrous, ≥ 99.9%). The precursor solution was magnetically stirred on a hotplate at 70 °C for 60 min to get the clear homogeneous solution. The electrode deposited SiO_2_/Si substrate was cleaned by two sonication processes in acetone and 2-propanol (isopropyl alcohol) for 20 min each round and dried with nitrogen flow. 50 µl of Cs_2_AgBiBr_6_ precursor solution was spin coated on the substrate at 1500 rpm for 60 s. After spinning, the material on the electrodes were carefully removed by cotton buds and followed by subsequent annealing at 250 °C for 5 min for the formation of phase pure double perovskite crystal structure.

### Cs_2_AgBiBr_6_ material characterization

The deposited thin-film X-ray diffraction data for Cs_2_AgBiBr_6_ were recorded using a Bruker D8 XRD machine. To avoid the crystal interference of the highly crystalline Si substrate, we used a soda lime glass with 1 mm thickness as the substrate for XRD measurements (Fig. 1b). SEM images for the film deposited on the SiO_2_/Si substrate were obtained using a Carl Zeiss instrument with an acceleration voltage of 2 kV for the top-view images and for studying the elemental composition using EDX spectrum (20 kV). All of the samples were pre-sputtered with carbon, and mirror and through-the-lens detectors were employed.

### Cs_2_AgBiBr_6_ FET characterization

The Cs_2_AgBiBr_6_ thin film FETs with different channel lengths were electrically characterized by using the computer interfaced Keithley 2602 under pulse mode measurement. The circuit connections along with the schematic of the FET are illustrated in Fig. 1c. The electrical contacts with the devices were made by using gold wirers and a portable probe station with spring-loaded contacts. All the electrical measurements were done under ambient conditions. The transfer curves were measured by sweeping the gate voltage from − 20 to 40 V by keeping the V_ds_ as constant at − 40 V.
